# Young adult self‐harm: The role of victimisation and polygenic risk in a population‐based longitudinal study

**DOI:** 10.1002/jcv2.70061

**Published:** 2026-01-21

**Authors:** Filip Marzecki, Isabelle Ouellet‐Morin, Helena M. S. Zavos, Sylvana Côté, Richard E. Tremblay, Jean‐Philippe Gouin, Massimiliano Orri, Michel Boivin, Marie‐Claude Geoffroy

**Affiliations:** ^1^ Institute of Psychiatry, Psychology & Neuroscience King's College London London UK; ^2^ Department of Psychiatry McGill University & Douglas Mental Health University Institute Montreal Quebec Canada; ^3^ School of Criminology & the Research Center of the Montreal Mental Health University Institute University of Montreal Montreal Quebec Canada; ^4^ School of Public Health University of Montreal Montreal Quebec Canada; ^5^ Department of Pediatrics and Psychology University of Montreal Montreal Quebec Canada; ^6^ Department of Psychology Concordia University Montreal Quebec Canada; ^7^ Department of Epidemiology, Biostatistics, and Occupational Health, School of Population and Global Health McGill University Montreal Quebec Canada; ^8^ Danish Research Institute for Suicide Prevention Mental Health Centre Copenhagen Copenhagen Denmark; ^9^ School of Psychology Laval University Quebec City Quebec Canada

**Keywords:** polygenic scores, self‐harm, victimisation, young adulthood

## Abstract

**Background:**

Victimisation has been associated with self‐harm (with or without suicidal intent), but little is known about this association during young adulthood—a distinct developmental period. Further, not all individuals who experience victimisation will later engage in self‐harm, suggesting the influence of other factors. The present study examined whether perceived victimisation is associated with self‐harm during young adulthood while adjusting for confounders such as peer victimisation and mental health difficulties in adolescence. Additionally, we investigated whether genetic susceptibility for mental health difficulties moderates this association.

**Methods:**

Participants were from the Quebec Longitudinal Study of Child Development (1998–2023); a population‐based birth cohort from the Canadian province of Quebec, with victimisation and self‐harm measures in young adulthood (sample 1; *N* = 1235), and who provided blood samples and had been genotyped (sample 2; *N* = 552). At 20 years old, they were asked how often they had experienced forms of victimisations in the past 12 months (e.g., insulted you, put you down in front of others) without specifying the perpetrator. At 20–25 years old, participants reported self‐harm. Polygenic scores (PGSs) for depression, attention deficit hyperactivity disorder (ADHD) and suicide attempt were calculated.

**Results:**

Overall, 17.2% (sample 1) participants reported self‐harm at age 20–25 years. Victimisation was associated with self‐harm (OR = 1.66, 95% CI = 1.45, 1.89, for each standard deviation increase in victimisation), even after adjusting for confounders, for example, victimisation and mental health in adolescence (OR = 1.53, 95% CI = 1.26, 1.86). In sample 2, PGSs for depression and suicide attempt (not ADHD) were associated with self‐harm as well as victimisation in young adulthood, and none moderated the association between victimisation and self‐harm.

**Conclusion:**

Regardless of genetic susceptibility indexed by PGSs, young adult exposed to victimisation are at higher risk of self‐harm. Continuing efforts to prevent victimisation beyond childhood/adolescence and into young adulthood may contribute to reducing self‐harm risk.

## Key Points

**What's known?**

- Victimisation by various perpetrators is associated with self‐harm across the lifespan, but little research has focused specifically on young adulthood (18–29 years).

**What's new?**

- Our findings expand on previous research, showing that victimisation in young adulthood is associated with self‐harm after controlling for confounding factors such as peer victimisation and mental health difficulties in adolescence. Young adults exposed to victimisation were more likely to self‐harm, regardless of genetic susceptibility indexed by polygenic scores.

**What's relevant?**

- Prevention programmes should not only focus on adolescent peer victimisation or intimate partner violence in adulthood, but should also target victimisation of young adults, in forms such as peer violence in higher education, workplace bullying or dating violence.

## INTRODUCTION

Victimisation—whether perpetrated by family members, peers, or romantic partners—involves the intentional use of force or power against another person, which often leads to harm (Butchart et al., 2014) and can take the form of physical aggression, verbal aggression and exclusion (Butchart et al., 2014). It is consistently associated with a higher risk of self‐harm (self‐injury with or without suicidal intent) (Moran et al., 2024) across the life‐course (Fedina et al., 2023; Geoffroy et al., 2016; Islam et al., 2020; Marzecki et al., 2024; McManus et al., 2022). For example, individuals who reported peer victimisation during adolescence (Geoffroy et al., 2016) or intimate partner violence across adolescence and adulthood (McManus et al., 2022) were more likely to concurrently report self‐harm.

Longitudinal studies, which follow individuals over time, can help clarify temporal associations while accounting for confounders that precede victimisation. Such studies have shown that adolescents, young adults and adults with histories of domestic violence in childhood, peer victimisation in childhood and/or adolescence, or dating violence in adolescence, are at greater odds of engaging in self‐harm (Brendgen et al., 2019; Castellví et al., 2017; Dillon et al., 2013; Fedina et al., 2023; Marzecki et al., 2024; McManus et al., 2022; Serafini et al., 2017). Some research has also linked concurrent cyber‐victimisation to self‐harm in English and Welsh young adults (Baldwin et al., 2021), and different forms of lifetime victimisation to self‐harm in American young adults (Fedina et al., 2023). Furthermore, young adults in India (Appachu, 2024) brought up difficulties in self‐acceptance, self‐esteem, and setting boundaries as consequences of peer victimisation.

Research typically combines young adults (18–29 years) with adolescent or older adult samples, and it remains unclear whether victimisation during young adulthood uniquely contributes to increased risk of self‐harm. This question is especially relevant given that self‐harm is prevalent in young adulthood (Marzecki et al., 2024; O’Connor et al., 2018) and is a concern due to its association with suicide death (Moran et al., 2024).

Young adulthood (sometimes called emerging adulthood; 18–29 years) marks the physical, psychological and social transition from adolescence to adulthood (Arnett et al., 2014; Bonnie et al., 2015; USAID, 2012). New challenges, such as increased independence, as well as instability in work life and romantic relationships, may trigger new mental health challenges, including self‐harm behaviours (Arnett et al., 2014; Bonnie et al., 2015). Recent trends may also make the transition more challenging than before, for example, the extension of education and lifespan, higher living costs in proportion to income, more rapid than ever changes in society (e.g., digitisation), and climate change (McGorry et al., 2024). The challenges faced by young adults are different to those faced by adolescents and adults and have their own implications for the risk of self‐harm (Arnett et al., 2014; McGorry et al., 2024).

Concerning the aetiology of self‐harm in young adulthood, social and psychological factors, as well as genetic vulnerabilities should be considered. Prior victimisation in adolescence, prior mental health difficulties, and social marginalisation based on sexually diverse identity contribute to the risk of self‐harm (Brendgen et al., 2019; Rogers & Taliaferro, 2020) and may do so beyond victimisation in young adulthood. Given that victimisation in adolescence has been shown to predict self‐harm in young adulthood (Brendgen et al., 2019; Castellví et al., 2017), those victimised in adolescence may either exhibit heightened vulnerability to further victimisation in young adulthood (i.e., sensitisation hypothesis) or display greater resilience to it (i.e., steeling hypothesis), for instances, from having received support during adolescence (Rutter, 2012).

Furthermore, twin research has consistently shown a moderate heritability (30%–55%) of individual differences in self‐harm behaviours (Lim et al., 2022; Marzecki et al., 2024). The emergence of genome‐wide association studies (GWAS) has shown that self‐harm is influenced by many genetic variants of small effects (Campos et al., 2020). For example, multi‐ancestry and European ancestry GWAS meta‐analyses identified 7 genes associated with suicidal self‐harm, otherwise referred to as suicide attempt (Docherty et al., 2023), and a European ancestry GWAS identified 4 genes associated with self‐harm regardless of intent (Campos et al., 2020). Results from GWASs allow researchers to construct polygenic scores (PGSs) in independent cohorts, which represent an individual's genetic predisposition to an outcome. PGSs for a range of mental health outcomes, including depressive symptoms, attention deficit hyperactivity disorder (ADHD) and suicide attempt, have been linked to the risk of self‐harm in UK adults (40–69 years;Lim et al., 2020).

The findings from twin and genomic studies infer that when considering development of self‐harm behaviours, both environmental exposures and genetic factors must be considered. In the present study, we consider PGSs for depression and ADHD in relation to odds of self‐harm, because they respectively represent two main groups of psychopathologies—internalising and externalising symptoms, known to relate to self‐harm (Commisso et al., 2023). We also consider a PGS for suicide attempt, due to its overlap with self‐harm regardless of intent, and because its GWAS is based on a larger sample size than that for self‐harm (Campos et al., 2020).

Genetic and environmental influences are unlikely to act independently. Genetic factors may exacerbate or buffer the contribution of social factors to behaviour, or vice versa (Kendler et al., 2010). This is known as gene‐environment interaction (GxE) and has been documented for several environments and mental health conditions (Kendler et al., 2010; Wendt et al., 2021). For example, female twins with greater genetic predisposition to depression were more likely to develop major depression following experience of a stressful event (e.g., assault, breakup/divorce) than female twins with lower genetic predisposition to depression (Kendler et al., 2010).

The evidence concerning GxE in the development of depressive symptoms remains mixed, even when similar environments, such as childhood adversity or lifetime traumatic events, are considered (Coleman et al., 2020; Gillett et al., 2022; Perret et al., 2023; Peyrot et al., 2018). This might be due to the heterogeneity of research design and statistical models employed to investigate GxE. One study conducted with adults from the UK Biobank (aged 40–69) found evidence of interaction between polygenic effects on suicidality and post‐traumatic stress in predicting suicidal ideation and attempt, but it did not investigate the moderating effect of experiencing and reporting traumatic events (Wendt et al., 2021). Post‐traumatic stress is an emotional, psychological and behavioural response to traumatic events, related to but not interchangeable with the experience of such events. Little other research has built on the recent advances in GWAS (i.e., increased samples and statistical power) to investigate interactions between PGSs and environments pertaining to self‐harm, and we identified none in young adults. Furthermore, the current literature focuses on trauma or traumatic events and not on more common types of victimisation such as verbal (e.g., being made fun of) and social (e.g., being ignored) victimisation.

In order to fill in the research gaps concerning victimisation during young adulthood uniquely contributing to increased risk of self‐harm, the aims of the present study were to: (1) test whether greater frequency of victimisation in young adulthood is associated with self‐harm and whether such association (If any) persists after controlling for potential confounders, including victimisation and mental health in adolescence, sexual diversity, and familial socioeconomic status (SES); (2) as first exploratory aim, to test whether the association between victimisation and self‐harm in young adulthood (if any) was moderated by having previously experienced victimisation in adolescence; (3) as second exploratory aim, to test a sex moderation of the association between victimisation and self‐harm, as some research has identified self‐harm to be more common in young women than men (Marzecki et al., 2024); (4) to investigate whether PGSs for depression, ADHD or suicide attempt moderate the association between victimisation and self‐harm in young adulthood. For the non‐exploratory aims (1 and 4) we hypothesised that: (1) the association between greater frequency of victimisation in young adulthood with self‐harm would be significant and would persist after controlling for confounders; (2) PGSs for depression, ADHD and suicide attempt would moderate the association between victimisation and self‐harm in young adulthood.

## METHOD

### Participants

Data were drawn from Quebec Longitudinal Study of Child Development (QLSCD; Orri et al., 2021), an ongoing prospective birth cohort study conducted by Institut de la Statistique du Québec. This study follows 2120 singletons born in 1997 and 1998 in the Canadian province of Quebec, except babies born extremely prematurely (earlier than 24 weeks) or later than 42 weeks, and those whose parents did not speak either English or French. The study received full ethical approval from the Ethics Committee of the Institut de la Statistique du Quebec, the Research Ethics Board of the CHU Sainte Justine Research Center, and the Montreal West Island Integrated University Health and Social Services Centre. Written informed consent was obtained from participants and/or their parents at each data collection. The QLSCD was broadly representative of the ethnic and socioeconomic makeup of Quebec at the time of inception (Orri et al., 2021). Data collection covered all regions except for Cree and Inuit territories and Indigenous communities due to differences in the socio‐cultural context and institutional services. Procedures and questionnaires are publicly available at https://www.jesuisjeserai.stat.gouv.qc.ca/.

The analyses were conducted on two analytical samples—the phenotypic sample (Sample 1) of participants who reported victimisation and self‐harm in young adulthood, and a subsample of participants who were also genotyped (Sample 2). Of the initial 2120 participants, 58.3% (*N* = 1235) were included in Sample 1 and 27.8% (*N* = 552) in Sample 2. Sample 1 is smaller than the initial cohort mostly due to: loss of contact or refusing or stopping to respond to questionnaires (Orri et al., 2021). Sample 2 is smaller due to subsampling for genetic data collection. Compared to the initial cohort, both analytical samples included a higher proportion of participants who were female, white, part of a family with sufficient household income, were brought up by two parents and whose mothers had lower postnatal depressive symptoms. For further information on samples and operationalisation of early life characteristics, see Supporting Information S1: Tables S1 and S2.

### Measures

#### Victimisation in young adulthood

At 20 years old, participants were asked ‘during the past 12 months, how many times has another person…’, followed by nine different victimisation experiences, including physical, verbal, relational, property attack and cyber types of victimisation, by an unspecified perpetrator, and rated them as: never, once or twice, more often (adaptation of the Self‐Report Victimisation Scale Ladd & Kochenderfer‐Ladd, 2002; see Supporting Information S1: Appendix S1). A standardised (mean = 0, SD = 1) sum was used as a composite score, with higher scores on this measure indicating greater frequency of victimisation. The Cronbach's alpha was 0.78.

#### Self‐harm

Self‐harm was reported at ages 20, 23 and 25 (see Supporting Information S1: Appendix S1). At age 20, participants were asked to respond ‘yes’ or ‘no’ to the following questions: ‘In the past 12 months, I tried to kill myself’ and ‘In the past 12 months, I tried to harm myself’. Participants who answered ‘yes’ to either question were coded as 1 (self‐harm).

At ages 23 and 25, participants were asked two questions ‘In the past 12 months, did you ever deliberately harm yourself but not mean to take your life?’, with response options: ‘never’, ‘rarely’, ‘quite often’, ‘very often’, as well as ‘In the past 12 months, how many times did you actually try to take your own life?’, with response options ‘never’, ‘once’, ‘more than once’. Participants who reported any deliberate self‐harm or suicide attempts (i.e., responses other than ‘never’) were coded as 1 (self‐harm).

#### Polygenic scores

A subsample of the participants provided blood samples at age 10, from which DNA was extracted with the Qiagen DlexiGene DNA kit. All the participants who provided blood samples and whose samples passed the quality control (see Supporting Information S1: Appendix S2) made up the analytical sample for the genetic analyses (*N* = 552). Their samples were genotyped using the Illumina Infinium PsychArray‐24 chip. PGSs were calculated as a sum of effect‐size‐weighted count of single‐nucleotide polymorphism variants significantly associated with a phenotype of interest on the genome‐wide level. The effect sizes were derived from an independent GWASs of depression, ADHD and suicidal self‐harm (Andlauer & Nöthen, 2020; Demontis et al., 2023; Docherty et al., 2023; Howard et al., 2019), with no overlap between their samples and the sample of this study. PGS‐Depression and PGS‐ADHD as they were both associated with self‐harm in UK Biobank (Lim et al., 2020) and on representing two broad domains of psychopathologies—internalising and externalising symptoms, respectively. The recent PGS‐Suicide Attempt was chosen, because it closely overlaps with the outcome in the present study as self‐harm (regardless of intent) includes suicide attempt. PGS were computed using PRSice v2.2.11 (Euesden et al., 2015), where they were clumped for linkage disequilibrium with SNPs LD: *r* ^2^ < 0.1, within 250 kb windows, as parameters. The PGS was regressed on 10 principal components and converted into standardised residuals (see Supporting Information S1: Appendix S2).

#### Confounders

Biological sex was coded as male or female from medical birth records. The remaining confounders were reported at ages 12, 13, 15, 17 and 23 years. Perceived peer victimisation in adolescence was measured at 12, 13, 15 and 17 with a modified version of the Self‐Report Victimisation Scale (Ladd & Kochenderfer‐Ladd, 2002). A standardised composite of the measures across adolescence was derived as a mean of a minimum three timepoints. Prior mental health difficulties, that is, internalising (e.g., depressive, anxiety symptoms) and externalising symptoms (e.g., conduct problems, ADHD) in adolescence were measured at 15 and 17 years using the Mental Health and Social Inadaptation Assessment (Côté et al., 2017). Standardised sums of the scores at ages 15 and 17 were averaged for internalising and externalising separately. Familial SES during adolescence was measured as an aggregate of annual gross income, parental education level, and occupational prestige and standardised score across adolescence (ages 12, 13, 15, 17) were averaged. Sexual orientation, transformed into a binary sexual diversity variable (sexually diverse vs. heterosexual), was reported at age 23. See Supporting Information S1: Appendix S1 for more detail on the measurement of the confounding variables.

### Statistical analyses

Our analyses addressing the non‐exploratory aims were pre‐registered (https://osf.io/xcbm9). All analyses were conducted with R (CR Team, 2012). In preliminary analyses, we used chi‐squared tests and *t*‐tests to describe differences in the early‐life characteristics of the participants included and excluded in the analytical samples 1 and 2. We also described frequencies and means of the variables included in the initial sample. We used chi‐squared tests to evaluate sex differences in self‐harm prevalence. Finally, we used univariable generalised linear models to test the association of the potential confounders with the outcome (self‐harm).

For aim 1, we investigated the associations between perceived victimisation in young adulthood and self‐harm in young adulthood, both without (model 1) and with (model 2) adjustment for confounders, such as peer victimisation and internalising/externalising symptoms in adolescence. Missing data on confounding factors (ranging from 0% to 20.4%) were imputed using multiple imputations with the mice package in R (Van Buuren & Groothuis‐Oudshoorn, 2011). Twenty datasets were created and combined—the number corresponding to the percentage of data missing, as per recommended practice (Blazek et al., 2021).

For aims 2 and 3, we conducted two exploratory phenotypic analyses. First, we tested whether adolescent victimisation moderated the association between young adulthood victimisation and self‐harm in young adulthood by adding an interaction term in a logistic generalised linear model (i.e., adolescent victimisation × young adulthood victimisation). Second, we tested whether biological sex moderated the relationship between victimisation and self‐harm in young adulthood, also by adding an interaction term to the model (i.e., sex × young adulthood victimisation).

Finally, for aim 4, we examined whether a higher PGS for depression, ADHD, or suicide attempts was associated with self‐harm in adulthood and/or moderated the relationship between victimisation in young adulthood and self‐harm. We first tested additive effects, including both PGS (either for depression, ADHD or suicide attempt) and victimisation in young adulthood as predictor variables, and self‐harm in young adulthood as the outcome. We then tested their joint, or interactive, effects. These analyses were conducted separately for each PGS.

## RESULTS

### Descriptive statistics

The variables of interest of the main analytical sample (1) are reported in Table 1. The prevalence of self‐harm between the ages of 20 and 25 years, was 17.2% (*N* = 255), with a significant difference between male (13.9%) and female (20.0%) participants, *X* ^2^(1) = 8.9, *p* = .002. The specific prevalence rates were 9.4% (*N* = 116) at age 20, 10.0% (*N* = 138) at age 23, and 7.5% (*N* = 100) at age 25. Sex differences were present at ages 20 and 23 (more prevalent for female participants) but were no longer observed at age 25 (see Supporting Information S1: Table S3). The number of missing responses can be found in Supporting Information S1: Table S4.

**TABLE 1:** Descriptive statistics of the variables of interest of the sample (based on maximum available *N* in sample 1).

Characteristics	Sample 1 *N* = 1,235^a^
Biological sex assigned at birth	
Female	710 (57.5%)
Male	525 (42.5%)
Sexual diversity (reported at 23 years)	
Heterosexual	829 (73.9%)
Sexually diverse	293 (26.1%)
*N* missing	113
Socioeconomic status composite 12–17 years	0.50 (−0.56, 0.74)
*N* missing	239
Education at 21 years	
Secondary school	73 (6.4%)
College (e.g., pre‐university, technical)	224 (19.6%)
University	454 (39.7%)
Other	18 (1.6%)
Not studying	374 (32.7%)
*N* missing	92

^a^ *n* (%); mean (interquartile range‐IQR).

*Source*: Data compiled from the final master file of the Québec Longitudinal Study of Child Development (1998–2023), Gouvernement du Québec, Institut de la statistique du Québec.

At age 20 years, 37.1% (*N* = 444) participants reported no victimisation in the past 12 months, whereas 42.0% (*N* = 505) experiencing it ‘once or twice’ and 20.9% (*N* = 251) reported occurring ‘often’. The most common type of victimisation reported by the participants at least once or twice was being ‘insulted’ by others (53.7%), following someone ‘ignoring you or pretending not to recognise or see you’ (24.8%). The least common type was being ‘forced to give someone something that belonged to you’ (3.5%). For more detail, see Supporting Information S1: Table S5.

### Phenotypic generalised linear models

#### Association between victimisation in young adulthood and self‐harm

As shown in Table 2, in the univariable models, all the confounders were significantly associated with greater odds of self‐harm between 20 and 25 (ORs = 1.45–2.49), while higher familial SES was significantly associated with lower odds of self‐harm (OR = 0.84, 95% CI = 0.72, 0.97).

**TABLE 2:** Associations between victimisation (20 years) and self‐harm in young adulthood (20–25 years) in logistic generalised linear models; analytical sample 1 (*N* = 1235).

	Unadjusted	Adjusted
	OR (95% CI)	OR (95% CI)
Victimisation in young adulthood	1.66^∗∗∗^ (1.45, 1.89)	1.53^∗∗∗^ (1.26, 1.86)
Peer victimisation in adolescence	1.45^∗∗∗^ (1.25, 1.67)	‐
Internalising in adolescence	1.71^∗∗∗^ (1.49, 1.97)	‐
Externalising in adolescence	1.46^∗∗∗^ (1.28, 1.67)	‐
Female sex	1.54^∗∗^ (1.16, 2.03)	‐
Sexual diversity	2.49^∗∗∗^ (1.86, 3.34)	‐
Familial SES	0.84^∗^ (0.72, 0.97)	‐

*Note*: For continuous predictors odd ratios compare individual who differ by one standard deviation of a predictor, whereas for categorical predictors it compares individuals at a particular level of the predictor to a reference level; Unadjusted estimate reflects a simple regression model where the exposure is a sole predictor variable, whereas adjusted estimates adjust for the effect of the confounders.

Abbreviations: SES, socioeconomic status; OR, odds ratio.

^∗^ *p* < .05.

^∗∗^ *p* < .01.

^∗∗∗^ *p* < .001.

*Source*: Data were compiled from the final master file of Quebec Longitudinal Study of Child Development (1998–2023), ©Gouvernement du Québec, Institut de la statistique du Québec.

The first aim was to test whether greater frequency of victimisation in young adulthood is associated with self‐harm and whether such association (If any) persists after controlling for potential confounders. Table 2 also shows that one standard deviation higher in victimisation in young adulthood was significantly associated with 1.66 times greater odds (95% CI = 1.45, 1.89) of self‐harm in young adulthood (step 1). The association remained significant when the confounders were accounted for in the analyses (step 2), whereby one more standard deviation in victimisation related to 1.53 (95% CI = 1.26, 1.86) times greater odds of self‐harm in young adulthood (see Table 2).

In post‐hoc analyses (not pre‐registered), we applied a stricter selection of variables for the multivariable model. First, we modified the outcome variable (self‐harm) to be a composite of self‐harm reported at 23 and 25 years only, so that the exposure reporting strictly precedes the outcome reporting. Second, we removed one confounder (sexual diversity) which was reported after the exposure. Third, we tested the association of the confounders with the exposure variable, and removed sex and SES from the model, as they were not associated with the exposure (see Supporting Information S1: Table S6). This stricter multivariable model revealed a weaker but significant adjusted association, whereby one more standard deviation in victimisation in young adulthood related to 1.29 (95% CI = 1.05, 1.58) times greater odds of self‐harm at 23–25 years old (see Supporting Information S1: Table S7).

**FIGURE 1:**
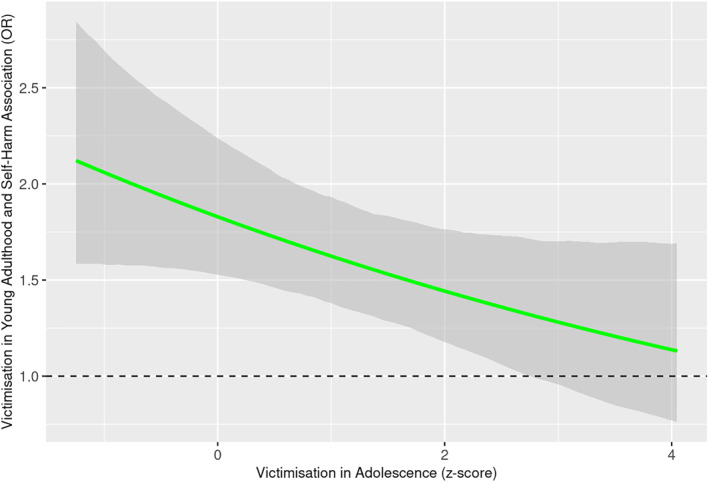
Interaction between young adulthood victimisation and adolescence victimisation in the association with self‐harm in young adulthood; analytical sample 1 (*N* = 1235). The green line shows the odds ratios (OR) for the association between standardised young adulthood victimisation and self‐harm (*y*‐axis) for each level of the adolescence victimisation *z*‐score (*x*‐axis), with accompanying 95% confidence intervals (grey area). OR = 0.89, 95% CI = 0.79, 0.99, *p* = .04. *Source*: Data were compiled from the final master file of Quebec Longitudinal Study of Child Development (1998–2023), ©Gouvernement du Québec, Institut de la statistique du Québec.

The second (exploratory) aim was to test whether the association between victimisation and self‐harm in young adulthood was moderated by having previously experienced victimisation in adolescence. We found that reporting peer victimisation in adolescence moderated the strength of the association between victimisation in young adulthood and self‐harm. As shown in Figure 1, participants who had reported more victimisation in adolescence had a weaker association between victimisation in young adulthood and self‐harm, whereas those who reported less victimisation in adolescence had a stronger association between victimisation in young adulthood and self‐harm (*β* = −.12; 95% CI = −0.23, −0.004; SE = 0.06; *p* = .04). See Supporting Information S1: Table S8 for more details.

#### Post‐hoc sex moderation analysis

The third (exploratory) aim was to test a sex moderation of the association between victimisation and self‐harm. Post‐hoc analyses (not pre‐registered) were conducted to investigate whether biological sex moderated the relationship between victimisation and self‐harm in young adulthood, and it did not (OR = 1.13, 95% CI = 0.86, 1.49). See the Supporting Information S1: Table S9 for details.

### PGS‐environment interaction generalised linear models

The fourth aim was to investigate whether PGSs for depression, ADHD or suicide attempt was associated with self‐harm in adulthood and/or moderated the association between victimisation and self‐harm in young adulthood.

#### Interaction between PGS‐depression and victimisation

We found that participants with a higher genetic vulnerability to depression indexed by PGS‐Depression, had greater odds of self‐harm in young adulthood (OR = 1.35 by each standard deviation, 95% CI = 1.11, 1.64). PGS‐Depression was also significantly associated with victimisation in young adulthood (*β* = 1.35, 95% CI = 1.11, 1.65; see Supporting Information S1: Table S10).

Victimisation and PGS‐Depression contributed independently to young adulthood self‐harm (step 2; additive model), suggesting that the participants' genetic vulnerability to depression did not affect the strength nor direction of the association between young adulthood victimisation and self‐harm (step 3; OR = 0.99, 95% CI = 0.80, 1.21; see Table 3 for details; see Supporting Information S1: Figure S1).

**TABLE 3:** Logistic generalised linear models predicting self‐harm in young adulthood (20–25 years) with standardised victimisation in young adulthood (20 years) and standardised PGS‐depression, PGS‐ADHD, or PGS‐suicide attempt; analytical sample 2 (*N* = 552).

		AIC (*df*)	OR (95% CI)
Step 1	Victimisation in young adulthood	527.10 (550)	1.67^∗∗∗^ (1.38, 2.03)
Step 2	Victimisation in young adulthood	522.70 (549)	1.66^∗∗∗^ (1.37, 2.02)
	PGS‐depression		1.33^∗^ (1.07, 1.66)
Step 3	Victimisation in young adulthood	524.70 (548)	1.67^∗∗∗^ (1.37, 2.03)
	PGS‐depression		1.33^∗^ (1.07, 1.67)
	Victimisation in young adulthood *PGS‐depression		0.99 (0.80, 1.21)
Step 2	Victimisation in young adulthood	528.80 (549)	1.68^∗∗∗^ (1.38, 2.04)
	PGS‐ADHD		0.94 (0.76, 1.17)
Step 3	Victimisation in young adulthood	530.70 (548)	1.68^∗∗∗^ (0.76, 1.18)
	PGS‐ADHD		0.95 (0.76, 1.18)
	Victimisation in young adulthood *PGS‐ADHD		0.96 (0.78, 1.18)
Step 2	Victimisation in young adulthood	523.10 (549)	1.66^∗∗∗^ (1.36, 2.01)
	PGS‐suicide attempt		1.32^∗^ (1.05, 1.65)
Step 3	Victimisation in young adulthood	525.20 (548)	1.66^∗∗∗^ (1.37, 2.03)
	PGS‐suicide attempt		1.33^∗^ (1.06, 1.67)
	Victimisation in young adulthood *PGS‐suicide attempt		0.97 (0.77, 1.21)

*Note:* For continuous predictors, odd ratios (OR) compare individual who differ by one standard deviation of a predictor, whereas for categorical predictors it compares individuals at a particular level of the predictor to a reference level.

Abbreviations: ADHD, attention deficit hyperactivity disorder; AIC, akike information criterion; PGS, polygenic score.

^∗^ *p* = .05.

^∗∗^ *p* = .01.

^∗∗∗^ *p* = .001.

*Source*: Data were compiled from the final master file of Quebec Longitudinal Study of Child Development (1998–2023), ©Gouvernement du Québec, Institut de la statistique du Québec.

#### Interaction between PGS‐ADHD and victimisation

We found that participants with higher levels of genetic vulnerability to ADHD, indexed by PGS‐ADHD, did not have greater odds of self‐harm in young adulthood (OR = 0.98; 95% CI = 0.81, 1.20). PGS‐ADHD was not significantly associated with victimisation in young adulthood (*β* = 0.98, 95% CI = 0.81, 1.21; see Supporting Information S1: Table S10). Additionally, this PGS did not significantly moderate the association between adult victimisation and self‐harm (step 3; OR = 0.96, 95% CI = 0.78, 1.18; see Table 3 and Supporting Information S1: Figure S1).

#### Interaction between PGS‐suicide attempt and victimisation

We found that participants with higher levels of genetic vulnerability to suicidal self‐harm, that is, suicide attempt, indexed by PGS‐Suicide Attempt, had greater odds of self‐harm in young adulthood (OR = 1.35; 95% CI = 1.11, 1.64). PGS‐Suicide Attempt was also significantly associated with victimisation in young adulthood (*β* = 1.35, 95% CI = 1.11, 1.64; see Supporting Information S1: Table S10). However, this PGS did not significantly moderate the association between young adulthood victimisation and self‐harm (step 3; OR = 0.97, 95% CI = 0.77, 1.21; see Table 3; see Supporting Information S1: Figure S1).

## DISCUSSION

This study examined the association between perceived victimisation and self‐harm specifically in young adulthood, in a prospective population‐based longitudinal study in Quebec, Canada. Young adults are overlooked in victimisation research by frequently being combined into samples of either adolescents or adults. However, this life stage is a distinct developmental period from adolescence (Arnett et al., 2014), marked by social changes that can increase the risk for poor mental health (McGorry et al., 2024).

We found that young adults who reported higher levels of victimisation were significantly more likely to engage in self‐harm between 20 and 25 years old (unadjusted OR = 1.66; adjusted OR = 1.53). These results were confirmed in a post‐hoc model where the outcome was reported at subsequent time‐points only, at 23 and 25 years old (unadjusted OR = 1.46; adjusted OR = 1.29). This finding extends those pertaining to different types of victimisations and self‐harm, whether they occurred in adolescence or adulthood (e.g., Brendgen et al., 2019; Islam et al., 2020; McManus et al., 2022). It builds upon the limited prior research focused on young adults, such as a cross‐sectional study linking lifetime victimisation reported in young adulthood with self‐harm (Fedina et al., 2023). By providing longitudinal evidence of this association, our study demonstrates its robustness, even after adjusting for key confounders. Finally, it extends the limited prior longitudinal research, such as a study on cyber‐victimisation and self‐harm in English and Welsh young adults (Baldwin et al., 2021), by showing that perceived victimisation in young adulthood, is associated with higher risk of self‐harm.

Victimisation was notably associated with self‐harm in young adulthood, even after controlling for confounding variables, including sociodemographic characteristics such as female sex, sexual diversity, and lower familial SES. Sex also did not moderate the relationship, suggesting that female and male participants who experienced victimisation had comparable risk of self‐harm. Importantly, we controlled for history of peer victimisation in adolescence, in line with the perspective of re‐victimisation (Brendgen et al., 2019), and the association in young adulthood was not explained by the effects of prior experiences of victimisation.

We found a significant moderating role of peer victimisation in adolescence, whereby those who experienced more peer victimisation in adolescence had a weaker association between victimisation and self‐harm in young adulthood. While, the moderation effect size was very small, it may suggest that some people who experience peer victimisation in adolescence also encounter opportunities to build resilience, which is in line with the steeling effect hypothesis of adversity, as opposed to the sensitising effect hypothesis (Rutter, 2012). This hypothesis suggests that exposure to adversity might contribute to strengthening individuals against exposure to later adversity, whereby they will be less negatively affected. Future research should explore this further. Additionally, we controlled for prior mental health difficulties, as an alternative explanation might be that individuals with a history of mental health problems are more likely to recall negative experiences (Francis et al., 2023), including victimisation, and be more likely to self‐harm. However, our analyses did not support this hypothesis. The association between perceived victimisation and self‐harm in young adulthood remained after controlling for internalising and externalising symptoms in late adolescence.

In the genetic analyses, perceived victimisation putatively impacted young people's risk of self‐harm regardless of genetic vulnerabilities indexed by PGSs for depression, ADHD and suicide attempt, as shown by interaction models. This may be evidence of a lack of GxE across the identified genetic variants in the relationship between perceived victimisation and self‐harm in young adulthood, which is in line with findings from the same cohort between PGS for depression and peer victimisation on depressive symptoms in adolescence (Perret et al., 2023). Some evidence suggests that those with genetic vulnerability to poorer mental health may be more likely to report adversity due to depression‐related cognitive processes. For example, adolescents with higher genetic predisposition to depression were more likely to report bullying victimisation, suggesting a gene‐environment correlation (rGE) pertaining to bullying (Schoeler et al., 2019). Our results suggests that the association between perceived victimisation and self‐harm remains even when accounting for rGE indexed by PGSs. Additive models showed that perceived victimisation was still significantly association with self‐harm in young adulthood after controlling for the effects of PGSs for depression and suicidal attempt. However, in all cases, the use of PGSs as indices of genetic vulnerability limits the validity of the conclusions concerning gene‐environment interplay, which we discuss in more detail in the limitations section (Pingault et al., 2022).

The novel results of our study, that victimisation in young adulthood is uniquely associated with self‐harm, emphasise the importance of interpersonal factors for a clinically significant sign of psychological distress (i.e., self‐harm) in young adulthood. Biological, psychological, and social changes all characterise development in young adulthood (Arnett et al., 2014) and our study points to the social as an important factor for psychiatric vulnerability. Interventions promoting positive relationships and prevention of victimisation in young adulthood would therefore contribute to lower rates of self‐harm, if the observed association is causal. For example, fostering social support may lead to fewer mental health difficulties including self‐harm in young adulthood (Scardera et al., 2020). Future research on psychological mechanisms may also provide insight to inform individual interventions. An investigation of psychological mediators would be an appropriate next step in the research on victimisation and self‐harm in young adulthood. A recent qualitative study identified self‐acceptance, self‐esteem, and boundary‐setting as struggles related to victimisation (Appachu, 2024). These malleable psychological factors could be tested as mediators, including in randomised controlled trials.

### Strengths and limitations

Strengths of this study include the longitudinal measures and extensive accounting for relevant confounding factors, including prior mental health difficulties and victimisation in adolescence, which reinforce our conclusions of a possible causal effect of victimisation on self‐harm. The QLSCD cohort was representative of the French‐ and/or English‐speaking majority of the province of Quebec, however, over time attrition of participants has led to overrepresentation of female participants, white participants and those of lesser socioeconomic disadvantage in the analytical sample, which may constrain the generalisability of the findings. Further to this, attrition may be related to other factors, such as mental health or experiencing adversity, which was not investigated here. Additionally, birth cohorts, such as the QLSCD, may become less representative of their generation over time, due to migration patterns, as they do not include individuals of the same age who migrated to Quebec after birth. This is a significant consideration as 13.5% of 15–29‐year‐olds in Quebec in 2016 were immigrants or non‐permanent residents born abroad (2019).

Further significant limitations pertain to the peer victimisation measure and the limitations of PGSs. Victimisation was self‐reported, meaning it could be an index of poorer wellbeing and not an objective measure of events. Those with a more positive life outlook might be more likely to forget negative experiences and not report them (Francis et al., 2023), whereas those with vulnerability to poorer mental health may be more likely to report adversity due to depression‐related cognitive processes. Therefore, our findings reflect a link between perceived victimisation and self‐harm, due to the exposure being self‐reported. However, past research showed a significant association between victimisation and self‐harm regardless of whether the exposure was self‐reported or reported by a family member (sibling or parent) with only small differences in effect sizes by informant, suggesting cross‐informant agreement between self and other‐reported measures (Baldwin et al., 2019).

The measure of victimisation used in our study weighs all forms of victimisation equally and does not capture sexual victimisation. Additionally, the prevalence of the physical victimisation was notably lower than of some of the social and verbal victimisation items. This limits the extent to which our findings are comparable with literature on physical and sexual victimisation.

Further, research on self‐harm regardless of intent is less applicable to the current diagnostic criteria, which delineate the behaviours into ‘suicidal behaviour disorder’ and ‘non‐suicidal self‐injury’, which differ from each other in prevalence and method, in addition to intention (Andover & Gibb, 2010). However, some people use non‐suicidal self‐injury to distract themselves from acting on suicidal intention (Edmondson et al., 2016) which illustrates the ambiguity and fluidity of intention during self‐harm episodes (Silverman et al., 2007). As such, whilst combining suicidal and non‐suicidal intention has limitations, so does delineating them.

PGSs are a limited index of genetic predisposition which only explain a small proportion of variance in behavioural outcomes and leave a substantial gap between said variance and heritability estimates (Pingault et al., 2022; Plomin & Von Stumm, 2022). PGSs carry the limitations of GWAS that they are based on, such as the lack of racial diversity in samples and sample sizes that are insufficient to detect significant but small effects (Blanc & Berg, 2020). Notably, in the case of the present study, the PGS‐ADHD was based on a relatively smaller sample size, deeming it less statistically powered to detect causal SNPs. Additionally, all PGSs were based on European‐ancestry samples and only white participants were genotyped, which restricts the conclusions of our genetically informed analyses to white participants. Finally, we report on sex differences, and it would be more appropriate to report on gender differences, because our investigation concerns social rather than biological mechanisms (e.g., potentially different associations between victimisation in self‐harm between two sexes). We used sex assigned at birth for the analyses because it was reported by a greater number of participants compared to gender identity. However, future studies should ideally use a well‐phenotyped gender variable for similar investigations.

## CONCLUSIONS

The putative association of perceived victimisation in young adulthood with the risk of self‐harm is itself of concern and is not explained through potential confounding factors, such as history of mental health difficulties, or re‐victimisation. Further, this association persist regardless of genetic vulnerability, as indexed by the PGSs tested. Social interactions play a significant role in the development of self‐harm behaviour in young adulthood, and interventions promoting positive relationships and preventing violence in young adulthood might help to prevent self‐harm in young adulthood.

## AUTHOR CONTRIBUTIONS

**Filip Marzecki**: Conceptualization; methodology formal analysis and visualization; data curation; original draft; reviewing and editing. **Isabelle Ouellet‐Morin**: Conceptualization; methodology; data curation; data funding acquisition and project administration; reviewing and editing. **Helena M. S. Zavos**: Conceptualization; supervision; reviewing and editing. **Sylvana Côté**: Conceptualization; data curation; data funding acquisition and project administration; reviewing and editing. **Richard E. Tremblay**: Data curation; data funding acquisition and project administration; reviewing and editing. **Jean‐Philippe Gouin**: Data curation; data funding acquisition and project administration; reviewing and editing. **Massimiliano Orri**: Conceptualisation; methodology; data curation; data funding acquisition and project administration; writing—reviewing and editing. **Michel Boivin**: Conceptualization; methodology, data curation; data funding acquisition and project administration; writing—reviewing and editing. **Marie‐Claude Geoffroy**: Conceptualization; methodology; formal analysis and visualization; data curation; data funding acquisition and project administration; supervision; writing—reviewing and editing.

## CONFLICT OF INTEREST STATEMENT

The authors declare no conflicts of interest.

## ETHICAL CONSIDERATIONS

The Quebec Longitudinal Study of Child Development (‘l’Étude longitudinale du développement des enfants du Québec’, or ELDEQ, in French) received the most recent update to its full ethical approval from the Ethics Committee of the Institut de la Statistique du Québec on 2nd December 2023 (document reference numbers: 9295‐18 and 3912‐18). The ethical evaluation documentation may be requested from the Institut de la Statistique du Québec. Written informed consent was obtained from participants and/or their parents at each data collection.

## Supporting information

Supporting Information S1

## Data Availability

Filip Marzecki had full access to all the data in the study and takes responsibility for the integrity of the data and the accuracy of the analyses. The data that support the findings of this study are available on request from the Research Data Access Point team of the Institut de la statistique du Québec. More information on how to access the data is available at https://jesuisjeserai.stat.gouv.qc.ca/informations_chercheurs/acces_an.html. The data are not publicly available due to privacy or ethical restrictions.
