# Supplementary material for: Young adult self‐harm: The role of victimisation and polygenic risk in a population‐based longitudinal study
Source: JCPP Adv. 2026 Jan 21;6(3):e70061. doi: 10.1002/jcv2.70061 (PMC13337135; doi:10.1002/jcv2.70061)
Supplement: Supplementary file 1 [file JCV2-6-e70061-s001.docx]

**Young Adult Self-Harm: The Role of Victimisation and Polygenic Risk in a Population-Based Longitudinal Study**

**Supporting Information**

**Appendix S1.** Measures

1. *Self-harm (outcome)*

At age 20, participants were asked to respond “yes” or “no” to the following questions: “In the past 12 months, I tried to kill myself” and “In the past 12 months, I tried to harm myself”.  Participants who answered "yes" to either question were coded as 1 (self-harm).

At ages 23 and 25, participants were asked two questions "In the past 12 months, did you ever deliberately harm yourself but not mean to take your life?”, with response options: “never”, “rarely”, “quite often”, “very often”, as well as “In the past 12 months, how many times did you actually try to take your own life?”, with response options “never”, “once”, “more than once”. Participants who reported any deliberate self-harm or suicide attempts (i.e., responses other than "never") were coded as 1 (self-harm).

2. *Victimisation in young adulthood (exposure)*

Participants were asked "In the past 12 months, how many times has another person…”, succeeding by the following victimisation experiences (rated on a 3-point scale – never, once or twice, more often):

“Insulted you”

“Put you down in front of others”

“Prevented you from being part of their group or team when you wanted to”

“Been physically rough with you (pushed you, hit you etc.)”

“Forced you to give them something that belonged to you”

“Made fun of you”

“Threatened you physically”

“Ignored you or pretended not to recognise or see you”

“Said bad things about you or threatened using email, chat room, cell phone, or social media”

3. *Peer victimisation in adolescence (confounder/moderator)*

A modified 6- or 7-item version of the Self-Report Victimization Scale (Ladd & Kochenderfer, 2002) was administered to participants at ages 12, 13, 15, and 17. They were asked “since the beginning of this school year, how many times did the following situation happen to you as school (rated on a 4-point scale; never, rarely – one or twice, often – about once a week on average, very often – more than once a week on average), succeeded by the following victimisation experiences:

“Someone called me names, insulted me or said mean things to me”

“Someone didn't let me be part of his or her group when I wanted to”

“Someone pushed, shoved, hit or kicked me”

“Someone said bad things behind my back to other students”

“Someone made fun of me, laughed at me”

“I was «taxed» by other students (someone made me pay them or give them something so they would leave me alone)”

Additionally, participants were asked a slightly different question regarding cyber-victimisation: “since the beginning of this school year, at school, how many times was I a victim of cyber-bullying (insults, threats, intimidation, etc.) on the internet or by cellphone (perpetrated by other students)”, which they rated on a 5-point scale (never, once, a few times, often, very often).

The scores at each time point (12, 13, 15, and 17 years) were averaged by deriving a mean of a minimum three timepoints, meaning that participants needed to have provided data at least three waves of data collection between 12 and 17 to have a mean peer victimisation in adolescence score. The mean score was standardised.

4. *Internalising and externalising in adolescence (confounders)*

At ages 15 and 17, the Mental Health and Social Inadaptation Assessment (Côté et al., 2017), assessed symptoms in the past 12 month on a 3-point Likert frequency scale (1 = never, 2 = sometimes, and 3 = often). The assessment includes 113 questions representing DSM-5 symptoms for a given disorder. For internalising conditions, it assesses social phobia, generalized anxiety, depression, and self-harm. For externalizing conditions, it assesses attention deficit hyperactivity disorder (ADHD) (three subscales: inattention, impulsivity, and hyperactivity), Conduct Disorder (four subscales: lying, stealing, breaking rules, and vandalism), psychopathy, Oppositional Defiant Disorder, aggression (four subscales: proactive aggression, reactive aggression, social aggression, and severe physical violence), and delinquency and contact with the police. For internalising symptoms, the depression and dysthymia problems, generalized anxiety problems and social anxiety problems subscales were used. A standardised sum of the scores of these subscales at age 15 and 17 was obtained and an average of age 15 and 17 was created for an adolescent internalising variable. For externalising, the oppositional or defiant problems, conduct problems, and ADHD symptoms subscales. A standardised sum of the scores of these subscales at age 15 and 17 was obtained and an average of age 15 and 17 was created for an adolescent externalising variable.

5. *Sexual diversity (confounder)*

At 23, participants responded to the following item: “People are different in their sexual attraction to other people. How do you identify yourself?” with the following answer options: “exclusively heterosexual”, “predominantly heterosexual, only incidentally homosexual”, “predominantly heterosexual, but more than incidentally homosexual”, “bisexual”, “predominantly homosexual, but more than incidentally heterosexual”, “predominantly homosexual, only incidentally heterosexual”, “exclusively homosexual”, “asexual, nonsexual”, and “unsure, questioning”. Participants were coded as sexually diverse if they selected any other option than “exclusively heterosexual”. This was then used as a binary variable.

**Appendix S2**. Genotyping for Polygenic Scores

1. *DNA collection and extraction*

DNA was extracted from blood samples with the Qiagen FlexiGene DNA kit Cat#5120. Next, PicoGreen DNA assay (Invitrogen Quant-iT™ PicoGreen™ dsDNA Assay Kit Cat#P7589) was used to test DNA concentration and purity.

2. *Genotyping & quality control*

Génome Québec created a custom chip based on the Illumina Infinium PsychArray-24v1.1 Beadchip to genotype participants. Exclusions were made based on missing of data higher than 5% (12), genetic duplicates (7), sex mismatches (4). SNPs were also excluded if they had a minor allele frequency (MAF) less than 1%, were in deviation from Hardy-Weinberg equilibrium (HWE) (p < 1×10-6), or had ambiguous strand information.

Genetic differences in the population can introduce bias, therefore population stratification was modeled using ten multidimensional scaling components, calculated on the pairwise genetic identity-by-state matrix, for which SNPs with HWE test p < 0.001 or a MAF <5% were excluded. The remaining SNPs were pruned using windows of 200 variants, a step size of 100, and a linkage disequilibrium threshold of r2<0.2. This process identified 134 genetic outliers, which were then excluded. Finally, 5 participants were excluded due to high autosomal heterozygosity.

3. *Imputation*

The 1000 Genomes Phase 3 reference panel was used to impute SNPs, using haplotypes from a reference panel stimated using SHAPEIT2 (Delaneau, Marchini, & Zagury, 2012). SNPs were imputed using IMPUTE2 (Howie, Donnelly, & Marchini, 2009) in 5 mega-basepair chunks with 500 kilobase buffers, using all reference data. Finally, variants with a MAF <1%, INFO metric <0.8 and HWE test p < 1×10-6 were excluded.

**Table S1**. Early-Life Characteristics at 5 Months of the Initial Sample, Sample 1, and Sample 2

| Characteristics | Initial Sample  N = 2,120*^1^* | Analytical Sample 1  N = 1,235*^1^* | Analytical Sample 2  N = 552*^1^* |
| --- | --- | --- | --- |
| Sex |  |  |  |
| Female | 1,040 (49%) | 710 (57%) | 331 (60%) |
| Male | 1,080 (51%) | 525 (43%) | 221 (40%) |
| Racialised ^a^ |  |  |  |
| Yes | 219 (10%) | 101 (8.2%) | NA ^b^ |
| No | 1,898 (90%) | 1,131 (92%) | NA ^b^ |
| Household Income Insufficiency at 5 months ^c^ |  |  |  |
| Sufficient | 1,571 (75%) | 984 (81%) | 455 (83%) |
| Insufficient | 511 (25%) | 237 (19%) | 93 (17%) |
| Single Parent Household |  |  |  |
| Yes | 171 (8.1%) | 74 (6.0%) | 22 (4.0%) |
| No | 1,949 (91.9%) | 1,161 (94.0%) | 530 (96.0%) |
| Maternal Educational Attainment ^d^ | | |  |
| High School Diploma or  Higher | 1,778 (84%) | 1,066 (86%) | 482 (87%) |
| No High School Diploma | 339 (16%) | 167 (14%) | 70 (13%) |
| Maternal age at birth | 29.3 (25.3, 33.0) | 29.6 (25.9, 33.1) | 29.6 (26.1, 33.2) |
| Maternal depressive symptoms ^d^ | 1.47 (0.56, 2.22) | 1.37 (0.56, 1.94) | 1.27 (0.28, 1.67) |
| Paternal depressive symptoms ^d^ | 0.32 (0.08, 0.42) | 0.31 (0.08, 0.42) | 0.31 (0.08, 0.42) |
| Child Internalising ^e^ | 0.91 (0.00, 1.00) | 0.90 (0.00, 1.00) | 0.88 (0.00, 1.00) |
| Child Externalising ^e^ | 5.08 (3.00, 7.00) | 4.94 (3.00, 7.00) | 5.10 (3.00, 7.00) |
| *^1^* n (%); Mean (IQR) | |  |  |

*Note*: Data were compiled from the final master file of Quebec Longitudinal Study of Child Development (1998-2023), ©Gouvernement du Québec, Institut de la statistique du Québec. ^a^ Defined as parents’ identification of the children to any of 11 non-white racialised categories. ^b^ Cell N < 5. ^c^ Insufficiency of income was defined as spending more than 20% of the annual income for basic needs, in addition to the average proportion spent by households of similar size and regional population density. ^d^ Assessed at 5 months maternal and paternal depressive symptoms assessed with twelve-item version of the Center for Epidemiological Studies-Depression rescaled to range 0-10 (Poulin et al., 2005); ^e^ Assessed at 29 months (17 months if 29 months was missing) using the Behavior Questionnaire mean scores (range 1-3) of 6 items for internalising and 10 items for eternalising (Collet et al., 2022)

*References*: Collet, O. A., Orri, M., Tremblay, R. E., Boivin, M., & Côté, S. M. (2022). Psychometric properties of the Social Behavior Questionnaire (SBQ) in a longitudinal population-based sample. International Journal of Behavioral Development, 01650254221113472.

Poulin, C., Hand, D., & Boudreau, B. (2005). Validity of a 12-item version of the CES-D [Centre for Epidemiological Studies Depression scale] used in the National Longitudinal Study of Children and Youth. Chronic Diseases and Injuries in Canada, 26(2-3), 65.

**Table S2**. Inferential Comparison of the Initial Sample, Sample 1, and Sample 2

| Characteristic | Initial Sample vs Sample 1 *^1^* | Initial Sample vs Sample 2 *^1^* | Sample 1 vs Sample 2 *^1^* |
| --- | --- | --- | --- |
| *Chi-squared test* | | | |
| Female vs Male | 21.91*** | 20.42*** | 0.86 |
| White vs Racialised ^a^ | 3.91* | 51.56*** | NA |
| Household Income Sufficient vs Insufficient ^b^ | 7.10** | 10.20** | 1.33 |
| Two Parent vs Single Parent Household | 4.66* | 10.28** | 2.64 |
| Maternal Education – No High School vs High School or Higher | 3.51 | 3.49 | 0.18 |
| *t-test* | | | |
| Maternal age at birth | -1.38 | -1.46 | -0.39 |
| Maternal depressive symptoms ^c^ | 2.09* | 3.21** | 1.50 |
| Paternal depressive symptoms ^c^ | 0.90 | 0.43 | -0.23 |
| Child internalising symptoms ^d^ | 0.05 | 0.37 | 0.31 |
| Child externalising symptoms ^d^ | 1.32 | -0.14 | -1.05 |
| *^1^* *t*; *χ*^2^; * = *p* < .05, ** = *p* < .01, *** = *p* < .001 | | | |

*Note*: Data were compiled from the final master file of Quebec Longitudinal Study of Child Development (1998-2023), ©Gouvernement du Québec, Institut de la statistique du Québec. ^a^ Defined as parents’ identification of the children to any of 11 non-white racialised categories. ^b^ Insufficiency of income was defined as spending more than 20% of the annual income for basic needs, in addition to the average proportion spent by households of similar size and regional population density. ^c^ Assessed at 5 months. Maternal and paternal depressive symptoms assessed with twelve-item version of the Center for Epidemiological Studies-Depression rescaled to range 0-10; ^d^ Assessed at 29 months (17 months if 29 months was missing) using the Behavior Questionnaire mean scores (range 1-3) of 6 items for internalising and 10 items for eternalising

**Table S3**. Sex Differences in Prevalence of Self-Harm at ages 20, 23, and 25 years (Analytical Sample 1)

|  | Prevalence – Male – n (%) | Prevalence – Female – n (%) | *X^2^* (df) | *p*-value |
| --- | --- | --- | --- | --- |
| 20 years | 6.38% | 11.69% | 9.73 (1) | .002 |
| 23 years | 8.20% | 12.04% | 4.71 (1) | .03 |
| 25 years | 7.07% | 7.80% | 0.16 (1) | .69 |

Note: Data were compiled from the final master file of Quebec Longitudinal Study of Child Development (1998-2023), ©Gouvernement du Québec, Institut de la statistique du Québec.

**Table S4.** Descriptive Statistics of the Victimisation Exposures (Mean, SD) and Self-Harm Outcomes (n, %) in the Initial Sample

| Characteristic | N = 2,120*^1^* |
| --- | --- |
| Adolescent peer victimisation (12-17 years) | 0.29 (0.14, 0.48) |
| Number missing | 872 |
| Adulthood victimisation (20 years) | 0.11 (0.00, 0.33) |
| Number missing | 885 |
| Victimisation at 20 years |  |
| Never | 444 (37.1%) |
| Once or twice | 503 (42.0%) |
| Often | 251 (20.9%) |
| Self-Harm at 20 years | 116 (9.4%) |
| Number missing | 885 |
| Self-Harm at 23 years | 138 (10.0%) |
| Number missing | 885 |
| Self-Harm at 25 years | 100 (7.5%) |
| Number missing | 885 |
| Self-Harm in young adulthood (20-25) | 255 (20.8%) |
| Number missing | 639 |
| *^1^* Mean (IQR); n (%) | |

*Note*: Data were compiled from the final master file of Quebec Longitudinal Study of Child Development (1998-2023), ©Gouvernement du Québec, Institut de la statistique du Québec.

**Table S5**. Prevalence of Different Forms of Perceived Victimisation at Age 20 (Analytical Sample 1)

| Form of Perceived Victimisation | Frequencies – N (%) | | | |
| --- | --- | --- | --- | --- |
|  | **Never** | **Once or twice** | **More Often** | **Not responded** |
| “Insulted you” | 577 (46.3%) | 518 (41.6%) | 140 (11.2%) | 10 (0.8%) |
| “Put you down in front of others” | 875 (70.3%) | 267 (21.4%) | 91 (7.3%) | 12 (1.0%) |
| “Prevented you from being part of their group or team when you wanted to” | 1124 (90.3%) | 92 (7.4%) | 19 (1.5%) | 10 (0.8%) |
| “Been physically rough with you (pushed you, hit you etc.)” | 1152 (92.5%) | 74 (5.9%) | 9 (0.7%) | 10 (0.8%) |
| “Forced you to give them something that belonged to you” | 1202 (96.5%) | 24 (1.9%) | 8 (0.6%) | 11 (0.9%) |
| “Made fun of you” | 940 (75.5%) | 237 (19.0%) | 58 (4.7%) | 10 (0.8%) |
| “Threatened you physically” | 1159 (93.1%) | 60 (20.2%) | 15 (3.7%) | 11 (0.9%) |
| “Ignored you or pretended not to recognise or see you” | 936 (75.2%) | 252 (20.2%) | 46 (3.7%) | 11 (0.9%) |
| “Said bad things about you or threatened using email, chat room, cell phone, or social media” | 1114 (89.5%) | 98 (7.9%) | 23 (1.8%) | 10 (0.8%) |

*Note*: Data were compiled from the final master file of Quebec Longitudinal Study of Child Development (1998-2023), ©Gouvernement du Québec, Institut de la statistique du Québec.

**Table S6**. Associations between Confounding Factors and Victimisation in Young Adulthood (20 years) in Generalised Linear Models; Analytical Sample 1 (*N* = 1,235)

|  | *Unadjusted* |
| --- | --- |
|  | ***β (95% CI)*** |
| Peer Victimisation in Adolescence | 0.39*** (0.34, 0.45) |
| Internalising in Adolescence | 0.84*** (0.68, 0.99) |
| Externalising in Adolescence | 1.33*** (1.07, 1.58) |
| Female sex | 0.00 (-0.12, 0.11) |
| Sexual Diversity | 0.19** (0.06, 0.32) |
| Familial SES | -0.03 (-0.09, 0.04) |

*Note*: Data compiled from the Québec Longitudinal Study of Child Development (1998-2023), Gouvernement du Québec, Institut de la statistique du Québec. * *p* < .05, ** *p* < .01, *** *p* < .001; Unadjusted estimate reflects a simple regression model where the exposure is a sole predictor variable

**Table S7**. Sensitivity Analyses of Associations between Victimisation (20 years) and Subsequent Self-Harm in Young Adulthood (23-25 years) in Logistic Generalised Linear Models; Analytical Sample 1 (*N* = 1,235)

|  | *Unadjusted* | *Adjusted* |
| --- | --- | --- |
|  | ***OR (95% CI)*** | **OR (95% CI)** |
| Victimisation in Young Adulthood | 1.46*** (1.27, 1.68) | 1.29* (1.05, 1.58) |
| Peer Victimisation in Adolescence | 1.41*** (1.2-, 1.66) | - |
| Internalising in Adolescence | 1.53*** (1.49, 1.97) | - |
| Externalising in Adolescence | 1.32*** (1.14, 1.54) | - |

*Note*: Data were compiled from the final master file of Quebec Longitudinal Study of Child Development (1998-2023), ©Gouvernement du Québec, Institut de la statistique du Québec. * *p* < .05, ** *p* < .01, *** *p* < .001; For continuous predictors odd ratios compare individual who differ by one standard deviation of a predictor, whereas for categorical predictors it compares individuals at a particular level of the predictor to a reference level; Unadjusted estimate reflects a simple regression model where the exposure is a sole predictor variable, whereas adjusted estimates adjust for the effect of the confounders

**Table S8.** Logistic Generalised Linear Model Assessing Adolescence Victimisation Moderation of the Association between Victimisation (age 20 years) and Self-Harm in Young Adulthood (ages 20-25 years); Analytical Sample 1 (N = 1,235)

|  | Victimisation | *OR (95% CI)* |
| --- | --- | --- |
| Step 1 | Young Adulthood | 1.66*** (1.45, 1.89) |
| Step 2 | Young Adulthood | 1.67*** (1.40, 1.99) |
|  | Adolescence | 1.13 (0.94, 1.35) |
| Step 3 | Young Adulthood | 1.83 ***(1.50, 2.23) |
|  | Adolescence | 1.19 (0.99, 1.43) |
|  | Young Adulthood*Adolescence | 0.89* (0.79, 0.99) |

*Note*: Data were compiled from the final master file of Quebec Longitudinal Study of Child Development (1998-2023), ©Gouvernement du Québec, Institut de la statistique du Québec. * *p* = .05, ** *p* = .01, *** *p* = .001; For continuous predictors odd ratios compare individual who differ by one standard deviation of a predictor (victimisation)

**Table S9.** Logistic Generalised Linear Model Assessing Sex Moderation of the Association between Victimisation (age 20 years) and Self-Harm in Young Adulthood (ages 20-25 years); Analytical Sample 1 (N = 1,235)

|  |  | *OR (95% CI)* |
| --- | --- | --- |
| Step 1 | Victimisation in Young Adulthood | 1.66*** (1.45, 1.89) |
| Step 2 | Victimisation in Young Adulthood | 1.67*** (1.46, 1.91) |
|  | Sex | 1.79*** (1.29, 2.47) |
| Step 3 | Victimisation in Young Adulthood | 1.37 (0.88, 2.15) |
|  | Sex | 1.72** (1.24, 2,40) |
|  | Victimisation in Young Adulthood * Sex | 1.13 (0.86, 1.49) |

*Note*: Data were compiled from the final master file of Quebec Longitudinal Study of Child Development (1998-2023), ©Gouvernement du Québec, Institut de la statistique du Québec. * *p* = .05, ** *p* = .01, *** *p* = .001; For categorical predictors odds ratio compares individuals at a particular level of the predictor to a reference level

**Table S10.** Generalised Linear Model Assessing Gene-Environment Correlation - the Associations between Standardised PGS-Depression, PGS-ADHD, or PGS-Suicide Attempt and Victimisation (age 20); Analytical Sample 2 (N = 552)

| Predictor (PGS) | *β (95% CI)* |
| --- | --- |
| PGS-Depression | 1.35** (1.11, 1.65) |
| PGS-ADHD | 0.98 (0.81, 1.20) |
| PGS-Suicide Attempt | 1.35** (1.11, 1.64) |

*Note*: Data were compiled from the final master file of Quebec Longitudinal Study of Child Development (1998-2023), ©Gouvernement du Québec, Institut de la statistique du Québec. * *p* = .05, ** *p* = .01, *** *p* = .001; For continuous predictors odd ratios compare individual who differ by one standard deviation of a predictor

**Figure S1.** Interaction between Victimisation in Young Adulthood and PGS-Depression (A), PGS-ADHD (B), or PGS-Suicide Attempt (C) in the Association with Self-Harm

A)	B)

C)

*Note:* The line shows the OR for the association between victimisation and self-harm (y-axis) for each level of a PGS (x-axis) for depression (A), ADHD (B), or Suicide Attempt (C), with accompanying 95% confidence intervals (grey area). Data were compiled from the final master file of Quebec Longitudinal Study of Child Development (1998-2023), ©Gouvernement du Québec, Institut de la statistique du Québec.
